# Study on the therapeutic effect of *Viola yedoensis* and *Leonurus japonicus* on bovine endometritis

**DOI:** 10.1093/jas/skaf332

**Published:** 2025-09-26

**Authors:** Xuewen Tan, Xingchen Wang, Nishang Liu, Huicong Li, Yingqiu Liu, Weimin Zhang, Shen Zhuang, Lin Ma, Yunpeng Fan

**Affiliations:** College of Veterinary Medicine, Northwest A&F University, Yangling 712100, P. R. China; Institute of Traditional Chinese Veterinary Medicine, Northwest A&F University, Yangling 712100, P. R. China; College of Veterinary Medicine, Northwest A&F University, Yangling 712100, P. R. China; Institute of Traditional Chinese Veterinary Medicine, Northwest A&F University, Yangling 712100, P. R. China; College of Veterinary Medicine, Northwest A&F University, Yangling 712100, P. R. China; Institute of Traditional Chinese Veterinary Medicine, Northwest A&F University, Yangling 712100, P. R. China; College of Veterinary Medicine, Northwest A&F University, Yangling 712100, P. R. China; Institute of Traditional Chinese Veterinary Medicine, Northwest A&F University, Yangling 712100, P. R. China; College of Veterinary Medicine, Northwest A&F University, Yangling 712100, P. R. China; Institute of Traditional Chinese Veterinary Medicine, Northwest A&F University, Yangling 712100, P. R. China; College of Veterinary Medicine, Northwest A&F University, Yangling 712100, P. R. China; Institute of Traditional Chinese Veterinary Medicine, Northwest A&F University, Yangling 712100, P. R. China; College of Veterinary Medicine, Northwest A&F University, Yangling 712100, P. R. China; Institute of Traditional Chinese Veterinary Medicine, Northwest A&F University, Yangling 712100, P. R. China; College of Veterinary Medicine, Northwest A&F University, Yangling 712100, P. R. China; College of Veterinary Medicine, Northwest A&F University, Yangling 712100, P. R. China; Institute of Traditional Chinese Veterinary Medicine, Northwest A&F University, Yangling 712100, P. R. China

**Keywords:** endometritis, inflammation, *Leonurus japonicus*, network pharmacology, *Viola yedoensis*

## Abstract

Endometritis in dairy cows has become a key challenge in the field of animal reproductive health. Currently, the primary treatment method is using antibiotics. However, antibiotics could induce pathogen resistance and the presence of drug residues. Traditional Chinese medicine has good advantages in the treatment of endometritis in dairy cows. This study aims to investigate the therapeutic effect of *Viola yedoensis* and *Leonurus japonicus* (TCMF) on endometritis in dairy cows. Firstly, LPS-induced cell inflammation model was established to evaluate the anti-inflammatory effects of TCMF, and then rat model of endometritis was constructed to preliminarily assess its therapeutic effects. Subsequently, based on mass spectrometry and network pharmacology, the mechanisms of TCMF in treating endometritis were investigated. Finally, clinical observations were performed to assess the therapeutic effects of TCMF on bovine endometritis. The results showed that TCMF significantly reduced IL-1β, IL-6, IL-8, and IL-18 mRNA levels compared to the LPS group (*P* < 0.001). Compared to the model group, TCMF significantly reduced the uterine index and bacterial load in rats (*P* < 0.05). Additionally, TCMF significantly reduced IL-1β, IL-6, and TNF-α protein levels (*P* < 0.001) and decreased the protein expression levels of TNF, PTGS2, and CASP3 (*P* < 0.01). Compared to the bovine endometritis group, TCMF significantly reduced the scoring of uterine discharge, the number of PMNs, and the bacterial load (*P* < 0.001), while increasing the levels of albumin (ALB) and superoxide dismutase in the blood (*P* < 0.01), the cure rate of endometritis in dairy cows could reach up to 80%. Metabolomics analysis revealed upregulation of cortisol in the serum of treated cows. These results indicated that *Viola yedoensis* and *Leonurus japonicus* had a positive therapeutic effect on endometritis, and exerted effect through TNF signaling pathway, which could replace the antibiotics in a certain extent.

## Teaser

*Viola yedoensis* and *Leonurus japonicus* possessed the better effect on treating endometritis in dairy cows, they can replace antibiotics, thereby reducing drug resistance and drug residues.

## Introduction

Bovine endometritis is an inflammatory disease of the uterus, diagnosed through endometrial cytology, typically characterized by neutrophil infiltration (Druker et al., 2022). This disease results in uterine smooth muscle damage and impaired uterine contractions, which can lead to early miscarriages and recurrent pregnancy losses (Pirtea et al., 2021). The economic impact of this disease is substantial, impairing reproductive efficiency through higher insemination costs, extended days open, and increased culling rates. Concurrently, systemic inflammation leads to reduced milk yield throughout lactation. Additional financial burdens include treatment expenses, veterinary services, and milk discard. Endometritis thus severely threatens dairy farm profitability by simultaneously compromising both reproductive and productive performance (LeBlanc, 2023). The causes of bovine endometritis are multifactorial, with microbial infections being the most common. Other contributing factors include improper feeding and management practices, as well as secondary diseases. Following parturition, the cow’s immune system is weakened, and the birth canal may be damaged. Insufficient care or improper procedures can facilitate the invasion of pathogenic microorganisms into the uterus, leading to endometritis.

Antibiotics remain the primary treatment for bovine endometritis, but their use promotes antimicrobial resistance (AMR), drug residues in products, and disruption of natural microbiota. These issues drive the need for sustainable alternatives. Traditional Chinese Medicine (TCM) presents a promising option, offering multi-target bioactive compounds that reduce inflammation, fight pathogens, and enhance immunity. With its holistic approach, TCM supports overall health and metabolic balance, rather than merely suppressing symptoms. This reduces dependence on antibiotics, limits drug residues, and helps curb AMR. Developing such natural treatments is essential for sustainable and publicly acceptable dairy farming. Luteolin, a natural flavonoid found in various plants, has been used to treat purulent Pasteurella multocida-induced endometritis in rats. In rats treated with both low and high concentrations of luteolin, the uterus appeared normal with only mild congestion and limited inflammatory cell infiltration (Zhang et al., 2022), indicating the promising therapeutic effects of luteolin in treating endometritis. Except single-ingredient treatments, the efficacy of TCM formulas in treating bovine endometritis has also been demonstrated. Study has shown that a medicine composed of *Phellodendron chinense*, *Scutellaria baicalensis*, *Carthamus tinctorius*, *Codonopsis pilosula*, and other herbs exhibited therapeutic effects comparable to oxytetracycline (OTC) after 10 and 21 days of treatment (Qian et al., 2020). Study formulated a prescription containing *Leonurus japonicus*, *Angelica sinensis*, *Ligusticum chuanxiong*, *Scutellaria baicalensis*, *Forsythia suspensa*, and *Zingiberis Rhizoma* for uterine infusion in dairy cows with endometritis. Compared to OTC, the high-dose and medium-dose TCM groups showed higher efficacy rates of 75% and 83.33%, respectively, exceeding the 66.67% efficacy rate of OTC (Sun, 2022).

Network pharmacology is an approach that integrates traditional medicine with information science, offering valuable insights into the mechanisms of action of TCM (Han et al., 2022). For instance, a network pharmacology analysis of the active components and potential targets of *Carthamus tinctorius L*. for treating endometritis identified 20 active ingredients and a total of 260 targets. By intersecting these with 1,007 disease-related targets, the ERα/PI3K/AKT signaling pathway was selected as a key therapeutic pathway. Experimental results in rats confirmed that this pathway is involved in the therapeutic effects of *Carthamus tinctorius* on endometritis (Chen et al., 2023).

In the present study, we first utilized an LPS-induced cell inflammation model to evaluate the anti-inflammatory effects of the TCMF and constructed a rat model of endometritis to preliminarily assess its therapeutic effects. Subsequently, based on network pharmacology, we explored the mechanisms of action of the TCMF in treating endometritis. Finally, clinical observations were performed to assess the therapeutic effects of the TCMF on bovine endometritis. This study provides theoretical support for the development of TCM-based treatments for bovine endometritis.

## Materials and Methods

### Statement of institutional animal care and use committee

This study was approved by the Animal Ethics Committee of Northwest A&F University and conducted following Laboratory animal—Guideline for ethical review of animal welfare in China (GB/T 35892-2018).

### Reagent

*Viola yedoensis* (the whole plant) and *Leonurus japonicus* (aerial parts) were purchased from Shaanxi Yikang Pharmaceutical Co., Ltd and were authenticated by associate professor Weimin Zhang of the College of Veterinary Medicine in Northwest A&F University. Yi mu sheng hua san (YMSHS, No. 161015148) was purchased from Xinxiang Huaxu Trade Co., Ltd; OTC (No. 120516200) was purchased from Hefei Qianfang Animal Health Technology Co., Ltd; Fetal Bovine Serum (F8318) was purchased from Sigma Co., Ltd; fluorescence quantitative kit, RNA extraction kit and reverse transcription kit were all purchased from Beijing Dining Biotechnology Co., Ltd; Rat ELISA kit was purchased from Shanghai Enzyme Linked Biotechnology Co., Ltd; Ceftiofur Hydrochloride Sodium (CEF, No. 20230801) was purchased from Zhengzhou Bai Rui Animal Pharmaceutical Co., Ltd; Diff-Quick Rapid Cell Staining Solution was purchased from Solarbio Co., Ltd.

### Cell and bacterial culture

Passage 9 (P9) bovine endometrial (BEND) cells were thawed from cryopreservation and cultured under standard conditions (37°C, 5% CO_2_). The bacteria were isolated from the uterine secretions of cows with endometritis by the Laboratory of Traditional Chinese Veterinary Medicine. Bacteria were then inoculated into LB medium and incubated overnight at 37°C. After adjusting the bacterial concentration to 1 × 10^9^ CFU/mL, all strains were mixed in equal proportions and stored for future use.

### Preparation of TCMF

The formula composed with *Viola yedoensis* (the whole plant) and *Leonurus japonicus* (aerial parts) was prepared, and the ratio of *Viola yedoensis* and *Leonurus japonicus* was 1:1 (w:w). The herbs were soaked overnight in a water-to-drug ratio of 1:10, then boiled for 1 h, with the process repeated twice. The decoction was filtered through gauze, then concentrated to a final concentration of 1 g/mL. The solution was stored at 4°C overnight to allow for sedimentation. Subsequently, the solution was centrifuged at 3,500 rpm for 15 min, and the supernatant was collected. The supernatant was then further filtered through filter paper to remove impurities and sterilized in a water bath at 100°C for 30 min. Finally, the solution was stored at 4°C.

### Effect of TCMF on anti-inflammatory of BEND cells

#### Cell viability

BEND cells were plated in 96-well plates at 5 × 10³ cells/well. Once the cells had fully adhered, the TCMF, YMSHS, and OTC were serially diluted to 10 concentrations, which were then added to the cells at final concentrations of 500, 250, 125, 62.5, 31.25, 15.63, 7.81, 3.91, 1.95, and 0.98 μg/mL. A Control group was also included, with four replicates per group. Following a 12 h incubation under standard culture condition, cell viability was quantified by MTT assay with absorbance readings at 570 nm (Multiskan™ FC Microplate Photometer, Thermo, USA).

#### Cell treatment

The cells were plated in 12-well plates at 8 × 10^4^ cells/well. After treatment with LPS at a concentration of 8 μg/mL for 12 h, the cells were then treated with the drug for an additional 12 h, followed by subsequent experiments.

#### Effect of TCMF on mRNA expression of inflammatory cytokines in BEND cells

Primers were designed using the NCBI website and synthesized by Sangon Biotech (Shanghai) Co., Ltd The primer sequences are provided in Table 1. The procedure was performed according to the instructions of the 2x Fast qPCR Master Mix (Green) kit.

**Table 1.** Primer sequences

Gene name	Sequences	Product length, bp
IL-1β	F: GCCTTGGGTATCAAGGACAA	128
	R: TTTGGGGTCTACTTCCTCCA	
IL-6	F: CAGATCCTGAAGCAAAAGATCGC	96
	R: CCCACTCGTTTGAAGACTGC	
IL-8	F: CATTCCACACCTTTCCACCC	106
	R: AGGCAGACCTCGTTTCCATT	
IL-18	F: CCAATGCTTTCAGCGCTCC	144
	R: AGCCATCTTTATGCCTGTGCTC	
TNF	F: GGACACCCAGAATGTGAGGG	102
	R: GGAGAGTTGAAGTCCACGCA	
CASP3	F: AGCGTCGTAGCTGAACGTAA	134
	R: TCCAAGGATATTCCAGAGTCCA	
PTGS2	F: AGTCTTTGGTCTGGTGCCTG	103
	R: GCCCCATTCTGGATGCTCTT	

### Therapeutic effect of TCMF on endometritis in rats

#### Animal and experimental design

SD rats were purchased from the Experimental Animal Center of the Medical School of Xi’an Jiaotong University. The rats were housed in a controlled environment with access to clean water and standard rat chow, under a 12 h light/12 h dark cycle. Fifty SD rats were acclimatized for 7 days and then randomly divided into 5 groups (10 rats per group): Control, Model, TCMF, YMSHS and OTC. Except for the Control group, each rat in the other groups received 200 μL of mixed bacterial suspension via uterine infusion (the mixed bacterial suspension was composed of equal proportion of *Escherichia coli*, *Streptococcus*, *Shigella*, and *Staphylococcus*, the concentration of the bacterial liquid for each type of bacteria was 1 × 10^9^ CFU/mL.), while the Control group was infused with 200 μL of sterile saline. This treatment was administered for 3 days. Afterward, rats in the TCMF and YMSHS groups were intragastrically administered 2.5 mL of the undiluted solution daily. Rats in the OTC group received 2.5 mL of a 0.2 g/mL solution daily. Rats in the Model and Control groups were given 2.5 mL of water daily. The treatments continued for 7 days. After the treatment period, the rats were euthanized, and blood was collected from the abdominal aorta. The uteri were then photographed, weighed, and stored at −80°C.

#### Bacterial load in rat uterus

The uterine cavity was gently rinsed with 1 mL of sterile saline repeated three times. The rinsing fluid was serially diluted 10-fold, and 100 μL of each dilution was inoculated onto blood agar plates. The plates were incubated at 37°C overnight, and the bacterial colonies on the plates were subsequently counted.

#### Histopathology examination

Uterine specimens were dissected, immersion-fixed in 4% paraformaldehyde (PFA), and paraffin-embedded. Sections were subjected to hematoxylin-eosin (HE) staining for morphological evaluation using light microscopy.

#### Detection of inflammatory cytokines mRNA expression in rat uterus

A 100 mg portion of uterine tissue was excised and minced, and then 1 mL of TRIzol reagent was added. The mixture was placed on ice and disrupted using an ultrasonic cell disrupter. Total RNA was extracted following the TRIzol Reagent instructions. RNA samples underwent reverse transcription using the Integrated First-Strand cDNA Synthesis Kit, according to the manufacturer’s instructions. T Subsequent qPCR amplification with 2✕ Fast Green Master Mix was performed in triplicate, with primer pairs detailed in Table 2. Relative gene expression quantification employed the comparative Ct method (2^−ΔΔCt^).

**Table 2.** Primer sequences

Gene name	Sequences	Product length, bp
TNF-α	F: TGGGCTCCCTCTCATCAGTT	100
	R: TCCGCTTGGTGGTTTGCTAC	
IL-6	F: CTCATTCTGTCTCGAGCCCAC	111
	R: GGCTGGAAGTCTCTTGCGGA	
IL-1β	F: TGGCAACTGTCCCTGAACTC	107
	R: AAGGGCTTGGAAGCAATCCTTA	
GAPDH	F: ACTCCACTCACGGCAAATTC	88
	R: TCTCCATGGTGGTGAAGACA	

#### ELISA

The collected blood was left at room temperature for 2 h to allow serum separation. Once separated, the sample was centrifuged at 3,500 rpm for 15 min, and the serum was collected. A 0.1 g portion of rat uterus was excised and placed in a centrifuge tube, followed by the addition of 0.9 mL of saline. The tissue was homogenized using a grinder. The sample was then centrifuged at 3,000 rpm for 10 min, and the supernatant was collected. Protein concentration was measured using a BSA kit and adjusted to a uniform level. The levels of relevant inflammatory cytokines (IL-1β, IL-6, and TNF-α) in both rat uterine tissue and serum were determined using ELISA kit.

### Analysis of chemical compositions of TCMF by mass spectrometry

The sample extracts were analyzed using a Waters Acquity I-Class PLUS UPLC system coupled with an Applied Biosystems QTRAP 6500+ mass spectrometer, equipped with a Waters HSS-T3 column (1.8 μm, 2.1 × 100 mm) and a mobile phase of solvent A (ultrapure water containing 0.1% formic acid and 5 mM ammonium acetate) and solvent B (acetonitrile with 0.1% formic acid). The gradient elution program initiated at 98% A/2% B (1.5 min), linearly shifted to 50% A/50% B over 5.0 min, then to 2% A/98% B by 9.0 min (held 1 min), followed by re-equilibration to initial conditions (98% A/2% B) within 1 min and maintained for 3 min, with a constant flow rate of 0.35 mL/min at 50°C column temperature and 4 μL injection volume, interfaced to an ESI-triple quadrupole-linear ion trap (QTRAP) MS detector.

The ESI source operated in polarity-switching mode (positive: 5,500 V; negative: −4,500 V) at 550°C with gas parameters (GS I:50 psi, GS II:55 psi, CUR:35 psi) and medium CAD, calibrated via 10/100 μmol/L polypropylene glycol standards in QQQ/LIT modes respectively, employing nitrogen-mediated MRM detection with compound-specific declustering potentials (DP) and collision energies (CE) dynamically optimized across chromatographic windows.

### Network pharmacology research

#### Network pharmacological target prediction

Based on mass spectrometry analysis, the identified compounds were input into the PharmMapper database (https://www.lilab-ecust.cn/pharmmapper/) and swisstargetprediction database (http://www.swisstargetprediction.ch/) to retrieve related targets. Gene annotations for the target proteins were obtained via cross-referencing in the UniProt database (https://www.uniprot.org/). Using the keyword “endometritis,” potential targets for the condition were searched in the GeneCards database (https://www.genecards.org/), DrugBank database (https://go.drugbank.com/), and DisGeNET database (https://www.disgenet.org/). The intersection between the drug potential targets and endometritis-related targets was identified. Intersecting targets were interrogated through the STRING database (https://cn.string-db.org/) to establish protein interaction networks, which were subsequently visualized and topologically analyzed using Cytoscape software with a confidence threshold >0.4. The Cytoscape results were further analyzed using the Metascape database (https://metascape.org/gp/index.html) for Kyoto Encyclopedia of Genes and Genomes (KEGG) pathway enrichment analyses. The final results were imported into Bioinformatics.com.cn (https://www.bioinformatics.com.cn/) to create an infographic. Core targets and their corresponding active ingredients were selected as receptors and ligands. Active ingredient structures (SDF format) and target protein coordinates (PDB format) were retrieved from PubChem (https://pubchem.ncbi.nlm.nih.gov/) and RCSB PDB databases (https://www.rcsb.org/) respectively, and molecular docking and visualization were performed using AutoDock, OpenBabel, and PyMOL software.

#### Target validation

The expression levels of relevant target mRNA were measured using the method described in “Effect of TCMF on mRNA expression of inflammatory cytokines in BEND cells” section. The primer sequences are provided in Table 1. Real-time PCR results were analyzed using the 2^−ΔΔCt^ method.

The expression of related proteins was detected using immunofluorescence. After the cells were treated according to the method described in “Cell treatment” section, Following 15 min fixation in 4% PFA at ambient conditions, cellular samples underwent sequential processing: 0.5% Triton X-100 permeabilization (10 min) and 2 h blocking with 5% BSA at 37°C. The prepared monoclonal antibody (1:250) was used as the primary antibody and incubated at 37°C for 2 h. Subsequently, FITC-labeled goat anti-rabbit IgG (1:200) was added and incubated at 37°C for 1 h. After washing with PBST three times, the results were observed under an inverted fluorescence microscope (ICX41, Ningbo Sunny Instrument Co., Ltd).

### Therapeutic effect of TCMF on clinical endometritis of dairy cows

#### Observation on clinical symptoms of dairy cows

The examination procedure, performed on multiparous cows (2–3 lactations) between 21 and 30 days postpartum, included visual inspection of the vulva and tail, rectal palpation, and vaginal examination. Healthy cows presented a pale red vulva with no discharge, a clean tail without secretion, and no fluid accumulation in the uterus upon rectal examination, with both uterine horns being of even thickness. Cows with signs of systemic disease, the temperature was >39.5°C, and uterine discharges characterized by a foul odor, reddish-brown color, or watery consistency were considered to have metritis (Sheldon et al., 2006). Meanwhile, the vaginal discharge score (VDS) was used to classify findings (Barragan et al., 2019): clear fluid corresponded to VDS 0 for healthy cows; VDS 1 was defined as <50% white purulent fluid; VDS 2, ≥50% white purulent fluid; VDS 3, red-brownish watery fluid without fetid smell; and VDS 4, fetid red-brownish watery fluid. Cows with VDS 1–4 and a polymorphonuclear leukocyte (PMN) proportion greater than 18% in uterine cytology were diagnosed with clinical endometritis.

#### Animal experiment

According to the above method, 10 healthy cows and 28 cows with endometritis were selected from the cattle farm, and the sick cows were randomly divided into 3 groups: Endometritis, TCMF and CEF. Control and Endometritis groups received 5 L of plain water daily, while the TCMF group received 5 L of water containing 500 g of TCMF; all administrations were delivered orally as a drench. CEF group received daily intramuscular injections of ceftiofur hydrochloride sodium at a dose of 2.2 mg/kg for a total of 7 days. The cure rate was assessed after the 7-day treatment period.

#### Characteristics of uterine secretion

Uterine secretions from the cows were collected and classified based on the color, proportion of purulent material, and odor. The classification system is as follows: 0 points for transparent fluid; 1 point for white purulent fluid with less than 50%; 2 points for white purulent fluid with more than 50%; 3 points for red-brownish watery fluid without fetid smell; and 4 points for fetid red-brownish watery fluid (Barragan et al., 2019).

#### Detection of polymorphonuclear leukocytes in uterine secretion

Uterine secretions from the cows were collected and centrifuged at 1,200 g for 10 min. Smears were prepared from the sediment and stained with Diff-Quick stain. If the proportion of PMNs was less than 5%, the cow was considered to have recovered.

#### Evaluation of therapeutic effect of cow uterus

After treatment, cows were re-examined to assess their response, which included rectal palpation and bacterial isolation. The absence of pathological fluid in the uterine cavity, coupled with a significant reduction in bacterial load, serves as evidence of clinical recovery from endometritis. The cure rate was calculated by dividing the number of cows that showed clinical recovery by the total number of cows treated in each group (Amin et al., 2023).

#### Detection of blood biochemical indexes in dairy cows

After treatment, blood samples were collected from the tail vein of the dairy cows, and the blood biochemical indices were analyzed following serum separation.

#### Non-targeted metabonomics analysis

Blood samples were collected from the tail vein of the dairy cows, put the sample in a centrifuge at 4 °C, centrifuge at 3,000 rpm for 10 min, take serum in an EP tube, and test the sample after a series of treatments. Chromatographic separation was conducted on a Thermo Fisher Vanquish UHPLC system interfaced with an Orbitrap Q Exactive HF-series mass spectrometer at Novogene (Beijing), employing a Hypersil Gold column (100 × 2.1 mm, 1.9 μm) with 0.2 mL/min flow under a 12 min gradient: 0–1.5 min 2% B (methanol/0.1% FA), 1.5–4.5 min 2–85% B, 4.5–10.5 min 85–100% B, 10.5–12 min 10–2% B, using water/0.1% FA as mobile phase A. MS detection operated in polarity-switching mode with optimized parameters: 3.5 kV spray voltage, 320°C capillary, 35 psi sheath gas, 10 L/min auxiliary gas, 60 S-lens RF, and 350°C auxiliary heater.

UHPLC-MS/MS raw data were analyzed through a semi-targeted workflow in Compound Discoverer 3.3 (Thermo Fisher), executing peak alignment/detection with QC-corrected quantification (5 ppm mass tolerance, 30% intensity tolerance, minimum intensity threshold) followed by total spectral intensity normalization. Molecular formula prediction via adduct/fragment ion patterns preceded cross-database annotation (mzCloud, mzVault, MassList) for compound identification. Statistical processing employed R-3.4.3/Python-2.7.6/CentOS-6.6 environments, implementing QC-based normalization (sample_raw/(Σsample/ΣQC1)) for non-Gaussian distributions and applying 30% QC CV filtration to finalize metabolite profiling.

### Data analysis

Statistical analyses were performed using IBM SPSS Statistics 21.0. Continuous variables are presented as mean ± SD. One-way ANOVA followed by Tukey’s post-hoc test for multiple comparisons was employed for inter-group comparisons; differences were considered statistically significant at *P* < 0.05, and significance levels are denoted as ^*^P < 0.05, ^**^P < 0.01, and ^***^P < 0.001. Significant differences between groups are indicated with different letters. Data and analytical results were visualized as bar graphs using GraphPad Prism 7.00.

## Results

### Effect of TCMF on anti-inflammatory of BEND cells

**Figure 1.**
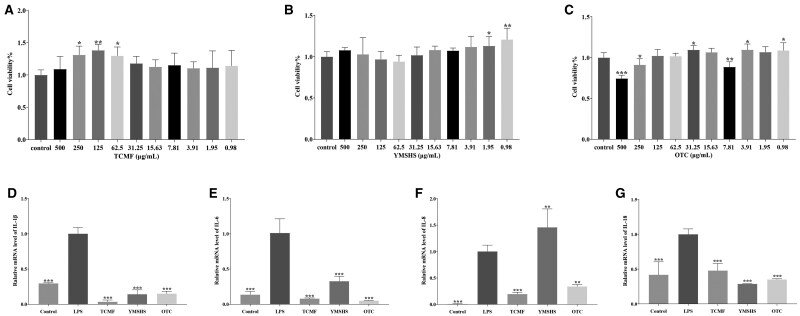
Effect of TCMF on anti-inflammatory of BEND cells. (A) Maximum safe concentration of TCMF; (B) Maximum safe concentration of YMSHS; (C) Maximum safe concentration of OTC; (D) mRNA expression of IL-1β; (E) mRNA expression of IL-6; (F) mRNA expression of IL-8; (G) mRNA expression of IL-18. Data are presented as the mean ± SD. * p<0.05, ** p<0.01, *** p<0.001.

The maximum safe concentration of the drugs was determined using the MTT assay, as shown in Figure 1. The TCMF significantly promoted cell growth at 125 μg/mL (*P* < 0.01) and at 250 and 62.5 μg/mL (*P* < 0.05) (Figure 1A). YMSHS significantly enhanced cell growth at 1.95 μg/mL (*P* < 0.05) and 0.98 μg/mL (*P* < 0.01) (Figure 1B). OTC significantly inhibited cell growth at 500 μg/mL (*P* < 0.001) and 7.81 μg/mL (*P* < 0.01), while also inhibiting cell growth at 250 μg/mL (*P* < 0.05). In contrast, OTC promoted cell growth at concentrations of 31.25, 3.91, and 0.98 μg/mL (*P* < 0.05) (Figure 1C). Based on these preliminary results, the final concentrations selected for subsequent experiments were 125 μg/mL for the TCMF, 500 μg/mL for YMSHS, and 31.25 μg/mL for OTC. The effects of these selected concentrations on inflammation in BEND cells were assessed by evaluating mRNA expression levels of inflammatory factors, as shown in Figure 1D–G. Compared to the control group, the LPS group exhibited significant increases in mRNA levels of IL-1β, IL-6, IL-8, and IL-18 (*P* < 0.001). Treatment with the TCMF, YMSHS, and OTC significantly reduced the mRNA levels of IL-1β, IL-6, and IL-18 (*P* < 0.001). Both the TCMF and OTC also significantly reduced IL-8 mRNA expression (*P* < 0.01). A significant difference was observed between the TCMF and YMSHS in IL-8 mRNA levels (*P* < 0.05), but no significant differences were found between the TCMF and OTC or between YMSHS and OTC in IL-1β and IL-18 mRNA expression (*P* > 0.05). These results suggest that both the TCMF and OTC exhibit the most effective anti-inflammatory effects in BEND cells, with YMSHS showing a comparatively weaker effect.

### Therapeutic effect of TCMF on endometritis in rats

**Figure 2.**
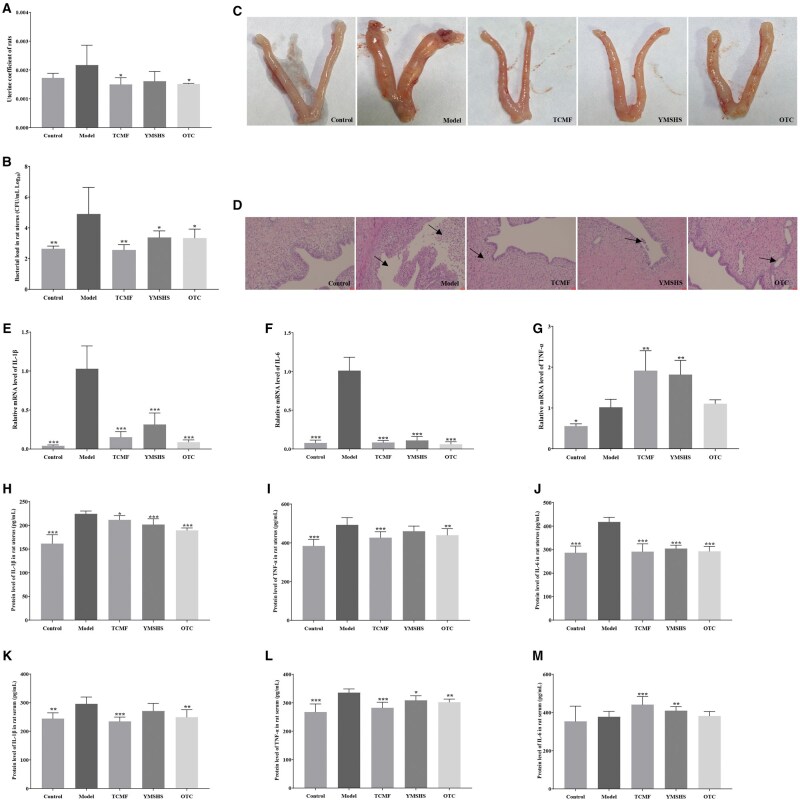
Therapeutic effect of TCMF on endometritis in rats. (A) Rat uterine coefficient; (B) Bacterial load in rat uterus; (C) Pictures of rat uterus; (D) Histopathological results of rat uterus; (E–G) mRNA level of inflammatory cytokines in rat uterus; (H–J) Protein level of inflammatory cytokines in rat uterus; (K–M) Protein level of inflammatory cytokines in rat serum. Data are presented as the mean ± SD. * p<0.05, ** p<0.01, *** p<0.001.

The results are shown in Figure 2A. Both the TCMF and OTC groups exhibited significant differences in uterine weight compared to the Model group (*P* < 0.05), while no significant difference was observed between the YMSHS group and the Model group. These findings suggest that both the TCMF and OTC reduced uterine weight in rats, while the Contorl group showed no significant difference from the Model group, likely due to the Contorl group being in estrus, which physiologically increases uterine size.

The bacterial load in rat uteri is shown in Figure 2B. Compared to the Control group, the Model group displayed a significant increase in bacterial load (*P* < 0.01). The TCMF group significantly reduced bacterial load compared to the Model group (*P* < 0.01), while both the YMSHS and OTC groups significantly decreased bacterial load (*P* < 0.05). Photographs of rat uteri (Figure 2C) revealed that the Control group had uniformly slender uteri with normal color. In contrast, the Model group exhibited significantly swollen and shortened uteri with congested uterine walls and a large amount of fluid in the cavity. The TCMF group showed slender, nearly uniform uteri with no significant congestion; the YMSHS group displayed mild swelling and shortening of the left uterus, with the right side remaining uniformly slender and no notable congestion; the OTC group showed mild swelling and shortening, with no significant congestion. The treatment outcomes are summarized in Table 3. The effective rate for the TCMF was 71.4%, with a cure rate of 42.9%. For YMSHS, the effective rate was 57.1%, with a cure rate of 28.6%. For OTC, the effective rate was 42.9%, with a cure rate of 14.3%.

**Table 3.** Treatment effects on rat endometritis

	Control	Model	TCMF	YMSHS	OTC
**Effective rate**	100%	0	71.4%	57.1%	42.9%
**Cure rate**	100%	0	42.9%	28.6%	14.3%

Histological examination of uterine tissues (Figure 2D) revealed that the Control group had a continuous, intact endometrium with a clean uterine cavity. In contrast, the Model group exhibited severe structural damage to the uterus, including extensive shedding of endometrial epithelium and a high number of neutrophils in the uterine cavity. The TCMF, YMSHS, and OTC groups all showed continuous and intact endometrial structures with minimal infiltration of inflammatory cells. The YMSHS group showed slight shedding of cells in the uterine cavity, while the OTC group exhibited significant endometrial hyperplasia. The mRNA levels of IL-1β, IL-6, and TNF-α in rat uteri were measured, and the results are shown in Figure 2E–G. Compared to the Control group, the Model group had significantly increased mRNA levels of IL-1β and IL-6 (*P* < 0.001), and TNF-α mRNA levels (*P* < 0.05). Compared to the Model group, the TCMF, YMSHS, and OTC groups all significantly reduced IL-1β and IL-6 mRNA levels (*P* < 0.001).

Regarding uterine tissue protein levels, the Model group exhibited significantly elevated levels of IL-1β, IL-6, and TNF-α compared to the Control group (*P* < 0.001). Serum IL-1β levels were significantly higher (*P* < 0.01), and TNF-α levels were markedly increased (*P* < 0.001) in the Model group. Compared to the Model group, the TCMF significantly reduced IL-1β protein levels in uterine tissue (*P* < 0.05) and significantly reduced TNF-α and IL-6 levels (*P* < 0.001). YMSHS significantly reduced IL-1β and IL-6 protein levels (*P* < 0.001), while OTC significantly reduced IL-1β, TNF-α, and IL-6 protein levels (P < 0.01). In serum, OTC significantly decreased IL-1β protein levels (*P* < 0.01), and the TCMF significantly decreased IL-1β levels (*P* < 0.001). YMSHS significantly reduced TNF-α protein levels (*P* < 0.05), the TCMF significantly reduced TNF-α levels (*P* < 0.001), and OTC also significantly reduced TNF-α levels (*P* < 0.01) (Figure 2H–M). These results indicate that the mixed bacterial solution can induce endometritis in rats, and that the TCMF, YMSHS, and OTC can effectively treat endometritis, with the TCMF showing the best therapeutic effect.

### Chemical components of TCMF

**Figure 3.**
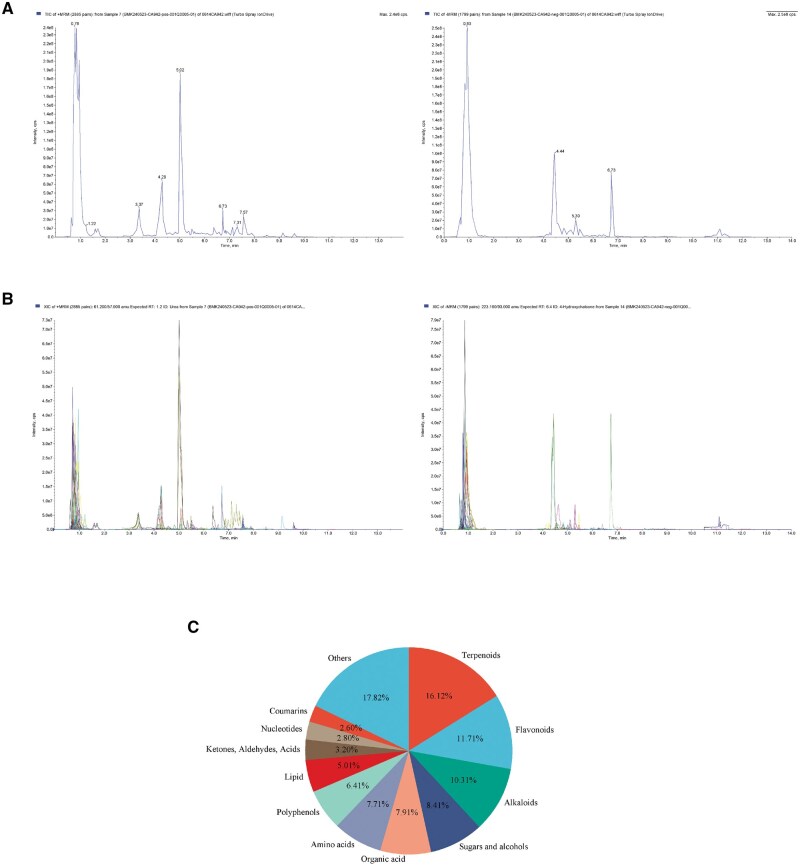
Chemical components of TCMF. (A) TIC of mixed phase mass spectrometry; (B) XIC spectrometry; (C) Proportion of compound categories in TCMF.

The active ingredients of the TCMF were analyzed, as shown in Figure 3A–C. Both TIC and MRM detection were performed in positive and negative ion modes, with qualitative and quantitative analysis conducted using the UPLC-Q TRAP-MS/MS system. The TIC diagram illustrates the ion intensity and retention time of each component’s metabolites in the chromatogram. In the XIC diagram, spectral peaks of different colors correspond to different metabolites. Using the UPLC-Q TRAP-MS/MS platform and the software database, a total of 2,079 chemical components were identified. These components were categorized into 12 major compound classes, including terpenoids, flavonoids, alkaloids, sugars and alcohols, organic acids, amino acids, polyphenols, lipids, and other compounds. As shown in Figure 3C, the most abundant components were terpenoids (16.12%), flavonoids (11.71%), and alkaloids (10.31%). Detailed information on compounds that constitute 1% or more of the total content is provided in Table 4.

**Table 4.** Identification of chemical constituents of TCMF

No.	Name	Class
1	Leonurine	Alkaloids
2	Dl-isocitric acid (trisodium salt)	Organic acid
3	Leonurine (hydrochloride)	Alkaloids
4	L-arginine	Amino acids
5	Dy131	Organic acid
6	Daphnin	Coumarins
7	Cichoriin	Coumarins
8	Stachydrine	Alkaloids
9	L-arginine (hydrochloride)	Amino acids
10	Malic acid	Organic acid
11	Pipecolic acid	Organic acid
12	Stachydrine hydrochloride	Alkaloids
13	Choline (chloride)	Lipid
14	Choline (bitartrate)	Ketones, aldehydes, acids
15	Ferric ammonium citrate	Others
16	Esculin	Coumarins
17	1-O-acetyl britannilactone	Terpenoids
18	Γ-aminobutyric acid	Organic acid
19	(S)-malic acid	Organic acid
20	D-melibiose	Sugars and alcohols
21	Alismol	Terpenoids
22	Sucrose	Sugars and alcohols
23	Isomaltose	Sugars and alcohols
24	Sodium citrate (dihydrate)	Ketones, aldehydes, acids
25	D-(+)-trehalose	Sugars and alcohols
26	2-Chloro-Dl-phenylalanine	Amino acids
27	Galactinol hydrate	Sugars and alcohols
28	D-proline	Amino acids
29	L-proline	Amino acids
30	Turanose	Sugars and alcohols
31	Pterolactam	Alkaloids
32	D-(−)-quinic acid	Organic acid
33	D-(+)-malic acid	Organic acid
34	L-ornithine (hydrochloride)	Amino acids
35	Lactulose	Sugars and alcohols
36	Sn-glycero-3-phosphocholine	Sugars and alcohols
37	3-Hydroxy-2-phenyl-propanamide	Alkaloids
38	Asperuloside	Terpenoids
39	Vasicinol	Alkaloids
40	Dl-3-phenylalanine	Amino acids
41	D-gluconic acid	Ketones, aldehydes, acids

### Network pharmacological target prediction

**Figure 4.**
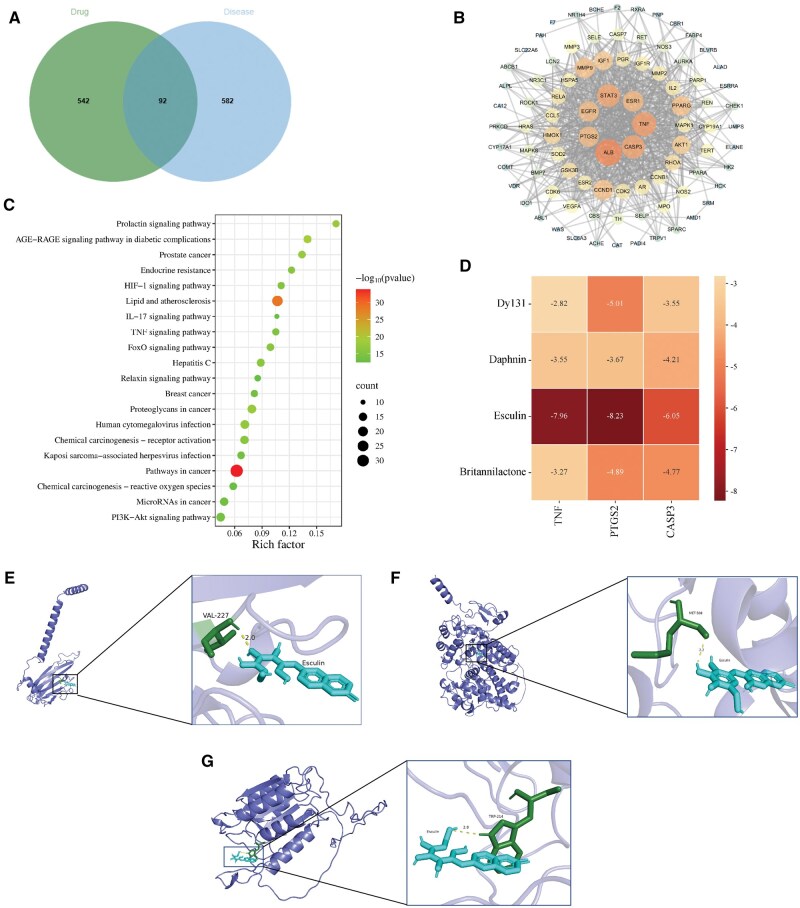
Network pharmacological target prediction. (A) Venn diagram of TCMF target and endometritis target; (B) PPI map of drug and disease targets; (C) KEGG enrichment analysis; (D) Molecular docking energy of target proteins with TCMF; (E–G) Active ingredient of TCMF and key target molecule docking diagram (E: TNF-Esculin; F: PTGS2-Esculin; G: CASP3-Esculin).

Based on the PharmMapper and SwissTargetPrediction databases, and disease-related databases, a total of 634 potential targets for the TCMF and 674 targets related to endometritis were predicted. Intersection of the key disease targets with those of the active ingredients revealed 92 common targets between the TCMF and the key targets for endometritis (Figure 4A). These intersecting targets were used to construct a protein-protein interaction (PPI) network via the String database, identifying core targets such as albumin (ALB), CASP3, TNF, ESR1, STAT3, EGFR, and PTGS2 (Figure 4B). The results were further analyzed in the Metascape database for KEGG pathway enrichment, as shown in Figure 4C. KEGG analysis suggests that the therapeutic mechanisms of the TCMF in treating endometritis may involve the TNF signaling pathway, IL-17 signaling pathway, and FoxO signaling pathway. Since CASP3, TNF, and PTGS2 are all involved in the TNF signaling pathway, subsequent research will focus on these three core targets and the TNF pathway. Molecular docking of the three core targets and their corresponding four major active ingredients was performed using Autodock software, with results shown in Figure 4D. All binding energies were negative, indicating that the active ingredients in the TCMF can spontaneously bind to the core genes. The active ingredient with the strongest binding affinity and its corresponding gene target were visualized using PyMOL software, as detailed in Figure 4E–G.

### Effect of TCMF on the mRNA and protein expression level of TNF signaling pathway

**Figure 5.**
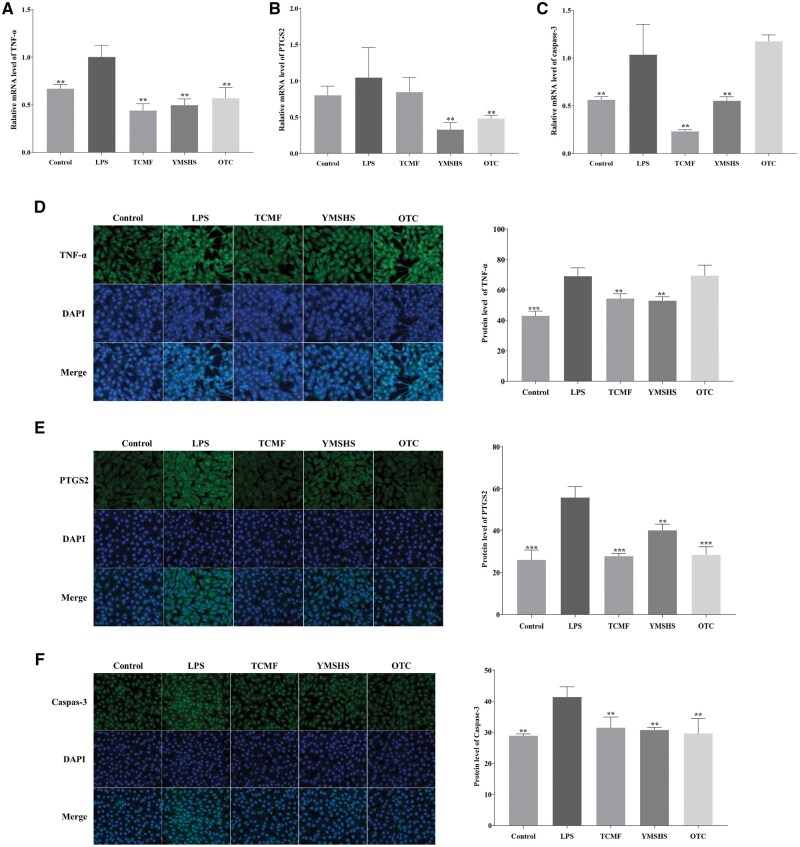
Effect of TCMF on the mRNA and protein expression level of TNF signaling pathway. (A) mRNA expression of TNF-α; (B) mRNA expression of PTGS2; (C) mRNA expression of caspase-3; (D) Protein expression of TNF-α (200×); (E) Protein expression of PTGS2 (200×); (F) Protein expression of caspase-3 (200×). Data are presented as the mean ± SD. ** p<0.01, *** p<0.001.

As shown in Figure 5A–C, the mRNA expression levels of TNF-α and caspase3 in the LPS group were significantly higher than those in the Control group (*P* < 0.01), while no significant difference was observed for PTGS2 mRNA expression. Compared to the LPS group, the TCMF and YMSHS groups significantly reduced the mRNA expression levels of TNF-α and caspase-3 (*P* < 0.01), whereas OTC significantly reduced TNF-α mRNA expression (*P* < 0.01). Both YMSHS and OTC significantly decreased PTGS2 mRNA expression. As shown in Figure 5D–F, the protein expression levels of TNF-α, PTGS2, and caspase-3 in the LPS group were significantly higher than those in the Control group (*P* < 0.01). The TCMF and YMSHS groups significantly reduced the protein expression levels of TNF-α, PTGS2, and caspase-3 (*P* < 0.01), while OTC significantly decreased the protein expression levels of PTGS2 and caspase-3 (*P* < 0.01). These results suggest that drug treatment of BEND cell inflammation may exert its effects through the TNF signaling pathway.

### Therapeutic effect of TCMF on clinical endometritis of dairy cows

**Figure 6.**
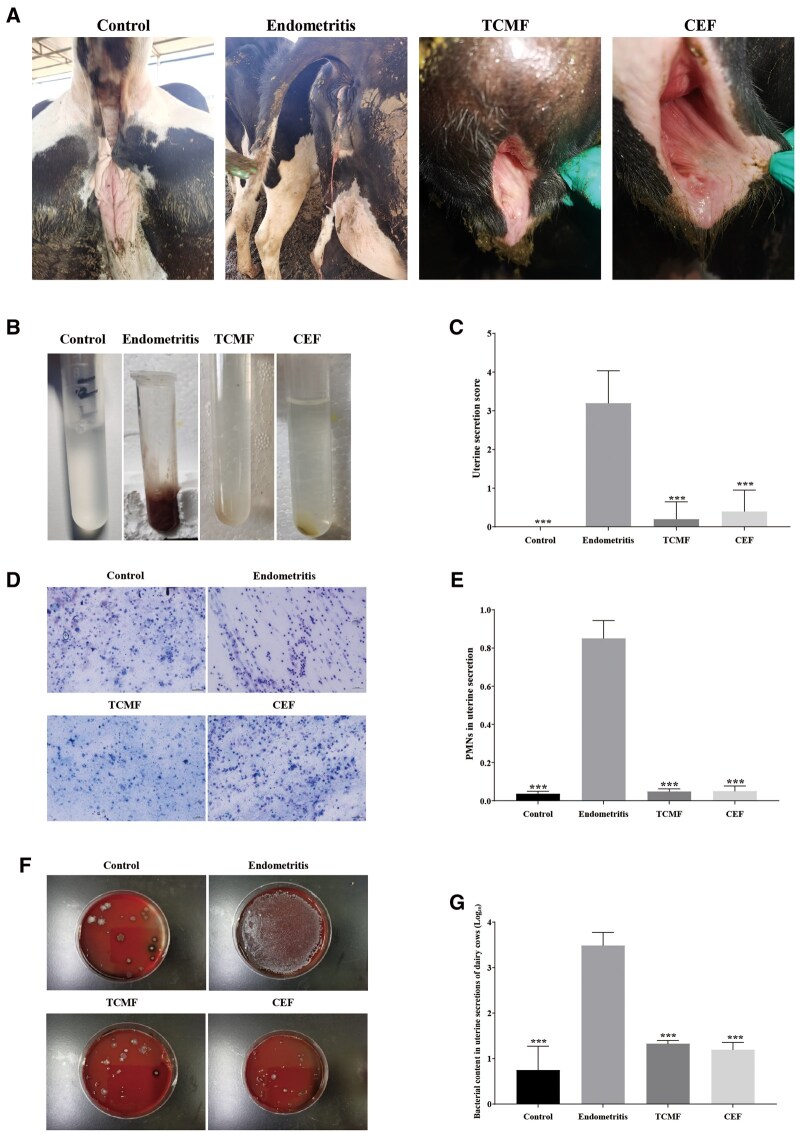
Therapeutic effect of TCMF on clinical endometritis of dairy cows. (A) Observation on clinical symptoms of dairy cows; (B, C) Uterine secretion score of dairy cows; (D, E) PMNs of uterine secretion; (F, G) Bacterial load in uterine secretion of dairy cows. Data are presented as the mean ± SD. *** p<0.001.

The results are shown in Figure 6A. In the Control group, no discharge was present on the cow’s vulva, the tail was clean, and rectal examination revealed that both uterine horns were uniform in size and consistent, with no noticeable fluctuation. In the Endometritis group, there was a large amount of red, foul-smelling discharge, with secretion adhering to the tail. Rectal examination showed that the uterine horns were hardened, uneven on both sides, and there was obvious fluctuation in the uterus. After pressure, a large amount of uterine discharge could be expelled. In the TCMF and CEF groups, most cows showed a significant reduction in uterine discharge, with it nearly disappearing, leaving only a small amount of dark brown dried discharge on the tail. Rectal examination revealed that the uterine horns regained elasticity, were of uniform size on both sides, and showed minimal fluctuation. However, some cows still expelled a small amount of uterine discharge, and there was residual discharge on the tail, with the uterine horns not fully restored and some fluctuation still present. In the Control group, the uterine discharge was clear, odorless, and transparent. In the Endometritis group, the uterine discharge was reddish-brown, viscous, and foul-smelling. In the TCMF and CEF groups, the uterine discharge was mostly clear and odorless, although some cows exhibited discharge containing less than 50% white pus. The uterine discharge score in the Endometritis group was significantly higher than in the control group (*P* < 0.001). Compared to the Endometritis group, both the TCMF and CEF groups showed significantly lower uterine discharge scores (*P* < 0.001) (Figure 6B, C). Cytological examination of the uterine discharge revealed the proportion of PMNs, as shown in Figure 6D, E. In the Control group, the main cells were sloughed uterine epithelial cells and stromal cells. In the Endometritis group, the majority of cells were neutrophils, with a small number of sloughed uterine epithelial and stromal cells. The TCMF and CEF groups mainly contained sloughed uterine epithelial cells and stromal cells. The PMN count in the Endometritis group was significantly higher than in the control group (*P* < 0.001), while both the TCMF and CEF groups significantly reduced the PMN count in the uterine discharge (*P* < 0.001). After bacterial culture of the uterine discharge, the Control group showed only a small number of bacterial colonies, whereas the Endometritis group exhibited a large number of bacterial colonies, significantly higher than the control group (*P* < 0.001). In the TCMF and CEF groups, the number of bacteria was significantly reduced (*P* < 0.001) (Figure 6F, G). Based on clinical examination and laboratory tests, the cure rate of uterine Endometritis in the TCMF group was 80%, while in the CEF group, it was 75% (Table 5). Compared to the Endometritis group, the TCMF and Control groups showed significant increases in ALB and superoxide dismu levels (*P* < 0.01) (Table 6).

**Table 5.** Treatment effects on cow endometritis

Group	Incidence number	Incidence rate	Cure number	Cure rate
Control	0	0	–	–
Endometritis	10	100%	0	0
TCMF	10	100%	8	80%^**^
CEF	8	100%	6	75%^**^

Data are presented as the mean ± SD. ** p<0.01.

**Table 6.** Blood biochemical indexes

	Control	Endometritis	TCMF	CEF
**ALB**	31.44 ± 1.01**	28.38 ± 2.01	31.98 ± 0.80**	30.12 ± 1.94
**SOD**	126.4 ± 12.40**	101.8 ± 13.97	134.6 ± 7.86***	131.2 ± 12.13**

Data are presented as the mean ± SD. ** p<0.01, *** p<0.001.

### Effect of the TCMF on serum metabolites of dairy cows

**Figure 7.**
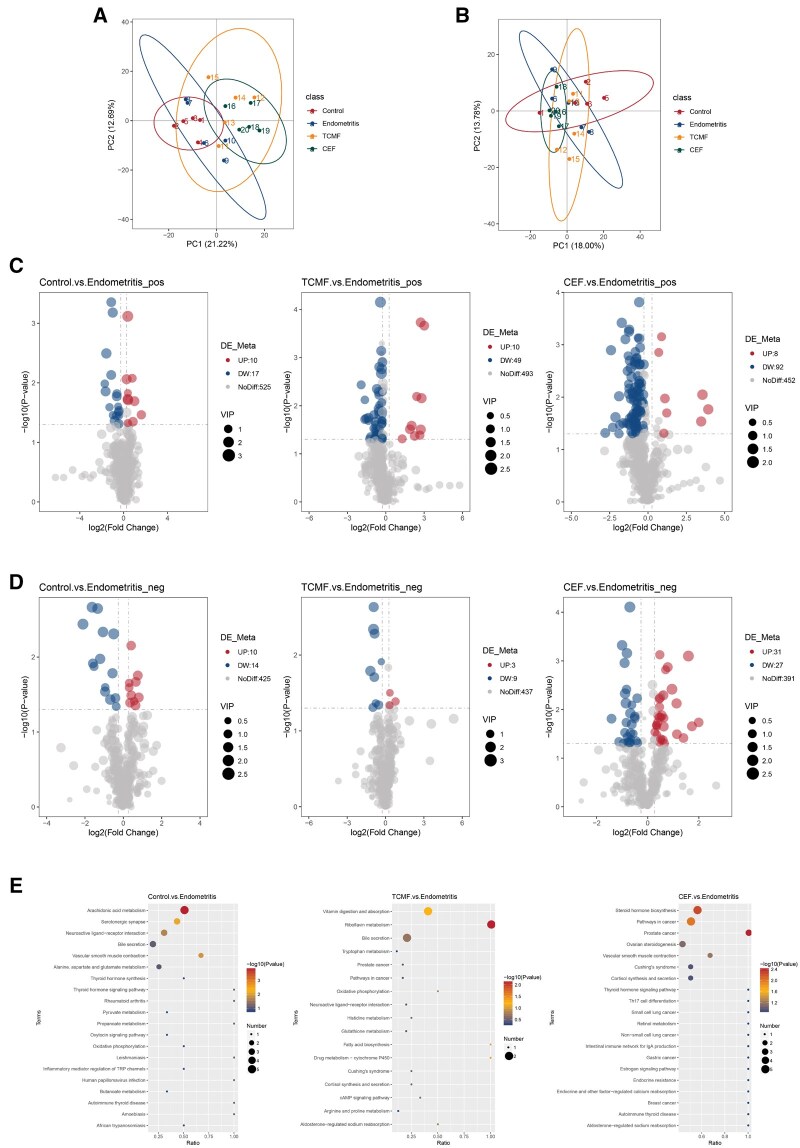
Effect of the TCMF on serum metabolites of dairy cows. (A, B) PCA map of differential metabolites; (C, D) The volcano and differential multiplier map of metabolites; (E) KEGG enrichment map of differentially metabolites.

Principal component analysis (PCA) analysis was performed on the peaks obtained from all experimental and QC samples. The smaller the differences in the QC samples, the better the stability of the method and the higher the data quality. This is reflected in the PCA plot, where the QC samples cluster together. As shown in the Figure 7A and B, the sample distribution is well-clustered, indicating good data quality. Volcano plots for the differential metabolites in the serum of the Control, TCMF group, CEF group and Endometritis group are shown in Figure 7C (positive ion mode) and Figure 7D (negative ion mode). In the positive ion mode, a total of 552 metabolites were compared. Compared to the Endometritis group, the Control group had 10 upregulated and 17 downregulated differential metabolites, the TCMF group had 10 upregulated and 49 downregulated differential metabolites and The CEF group had 8 upregulated and 92 downregulated differential metabolites. In the negative ion mode, a total of 449 metabolites were compared. Compared to the Endometritis group, the Control group had 10 upregulated and 14 downregulated differential metabolites, the TCMF group had 3 upregulated and 9 downregulated differential metabolites and The CEF group had 31 upregulated and 27 downregulated differential metabolites. The KEGG enrichment analysis between the Control and Endometritis groups revealed that the main pathway involved was arachidonic acid metabolism, while the differential metabolites of Control vs. Endometritis and CEF vs. Endometritis related to cortisol synthesis and secretion pathways (Figure 7E).

## Discussion

IL-1β, a central mediator of innate immune responses, orchestrates pathogen defense and clearance through its proinflammatory cytokine activity. However, in addition to its positive effects, IL-1β also has negative consequences, as it can promote acute tissue damage during the inflammatory process (Weber et al., 2010; Lopez-Castejon and Brough, 2011). IL-6 is a cytokine with dual functions, playing roles in both pro-inflammatory processes and immune response regulation, making it a “double-edged sword” (Linge et al., 2022; Zheng et al., 2022). IL-8, a chemokine for neutrophils, plays an important role in promoting inflammation (Russo et al., 2014; Zhu et al., 2021; Caetano et al., 2023). Therefore, controlling IL-8 expression is significant for anti-inflammatory effects. IL-18 induces the expression of IFN-γ during inflammation and, as a final product of pyroptosis, can cause severe tissue damage, thereby contributing to inflammation (Luan et al., 2022; Tang et al., 2022). In this study, we induced inflammation in BEND cells with LPS and treated them with four drugs. The results showed that the mRNA levels of IL-1β, IL-6, IL-8, and IL-18 were significantly reduced in the drug treatment groups compared to the LPS group, which is consistent with results from other studies using TCMF treatments for endometritis. One study using Biochemical Decoction to treat LPS-induced endometritis found that IL-1β, IL-6, and IL-8 levels were significantly reduced in the treatment group compared to the LPS group (Li et al., 2023). Additionally, quercetin has been shown to significantly inhibit LPS-induced IL-1β and IL-6 mRNA expression levels (Zhang et al., 2023). Since IL-1β and IL-18 are the final cytokines in the pyroptosis pathway (Latz et al., 2013), the significant inhibition of LPS-induced IL-1β and IL-18 mRNA levels by TCMF suggests that their anti-endometritis mechanisms may be related to pyroptosis, which warrants further investigation.

In the previous study, we isolated *E. coli*, *Staphylococcus*, *Streptococcus*, and *Shigella* from dairy cows with endometritis. Bacterial culture of uterine discharge from cows with endometritis revealed that the detection rate of *Staphylococcus* was significantly higher than that of other bacteria (Mekibib et al., 2024), suggesting that *Staphylococcus* plays a pathogenic role in the development of endometritis. Other studies have shown that within 24 to 48 h of endometritis onset, the content of *Shigella* significantly increases, only to decrease back to baseline by 72 h, while the content of *Streptococcus* also gradually rises in the early stages of infection (Li et al., 2024). This suggests that *Shigella* and *Streptococcus* may play a role in the early stages of infection. In this study, the rat model of endometritis was induced by intrauterine inoculation with a mixture of *E. coli*, *Staphylococcus*, *Streptococcus*, and *Shigella*. After modeling, rats showed significant uterine swelling, congestion of the uterine wall, and a large amount of fluid accumulation in the uterine cavity, indicating successful modeling. Bacterial culture of uterine lavage fluid revealed that the bacterial load in the uterus of rats in both the Control and treatment groups was significantly lower than that in the Model group, suggesting that the drug has therapeutic effects on rat endometritis. This is consistent with the previous research. (Demirel et al., 2019). However, the bacterial load in the Control group was not as low, possibly because healthy rats already harbor bacteria in their uterus, similar to the previous research (Mun et al., 2022), where the bacterial concentration in healthy mice’s uterus was found to reach 100 CFU/mL. In this study, a novel formula was designed and compared with the clinically widely used TCMF, YMSHS, and the OTC, to explore the therapeutic effects of the TCMF on endometritis. The results showed that the TCMF significantly alleviated the symptoms of endometritis, reduced epithelial sloughing in the uterus, and significantly lowered the mRNA levels of IL-1β and IL-6 in uterine tissue. It also significantly reduced the protein levels of IL-1β, IL-6, and TNF-α in uterine tissue, and IL-1β and TNF-α in serum, which is similar to the effects of Catalpol (Zhang et al., 2019). However, no similar trends were observed for the protein levels of IL-6 in serum and the mRNA levels of TNF-α. This may be because IL-6 is a unique inflammatory factor that plays a dual role in inflammation and immune regulation (Tanaka et al., 2014). The mRNA expression levels and corresponding protein levels may not always align, possibly due to various regulatory mechanisms during protein synthesis (C and M, 2020).

In this study, UPLC-ESI-MS/MS analysis was used to identify the active components of TCMF, resulting in the identification of 2,079 components. Of these, 41 components had a content greater than 1%. Based on these components, network pharmacology analysis was performed to predict the potential mechanisms of the TCMF in treating endometritis. The key targets TNF, PTGS2, and CASP3 were primarily concentrated in the TNF signaling pathway. These key targets are involved in the rapid development of endometritis, particularly during the inflammatory response, and their associated receptor factors can also induce apoptosis (Kalliolias and Ivashkiv, 2016). TNF-α and PTGS2 are major pro-inflammatory cytokines that play important roles in the onset and progression of many diseases. TNF-α, a pivotal early-phase mediator of inflammatory processes, orchestrates the activation of diverse intracellular signaling pathways, while PTGS2 is a key enzyme in regulating the production of PGE (Wang et al., 2023; Nazar et al., 2024). Research has shown that quercetin can inhibit the production of the pro-inflammatory factors TNF-α and PTGS2 in mouse uterine tissue and BEND cells induced by LPS, thereby improving the inflammatory response (Jiang et al., 2021). Caspases, a family of proteins closely related to eukaryotic cell apoptosis, include the most important member, CASP3, which plays a key regulatory role in multiple apoptosis pathways (Eskandari and Eaves, 2022). Studies have shown that astaxanthin significantly reduces Cleaved-Caspase-3 and alleviates LPS-induced apoptosis in BEND cells (Wan et al., 2020). Additionally, KEGG enrichment analysis indicates that the therapeutic effect of the TCMF on endometritis is primarily attributed to the TNF signaling pathway. In this study, after LPS treatment of BEND cells, the mRNA expression levels of TNF-α and caspase 3 were significantly increased. Upon treatment with the TCMF, the mRNA expression levels of TNF-α and caspase-3 were significantly reduced compared to the LPS group. On the protein level, LPS induced significant increases in the expression levels of TNF, PTGS2, and caspase-3 in BEND cells. However, after treatment with the TCMF, the expression levels of TNF-α, PTGS2, and caspase-3 were significantly decreased. These results suggest that the TCMF can alleviate LPS-induced uterine inflammation in cows and inhibit apoptosis triggered by inflammation-related cytokines.

Bovine endometritis is categorized into acute, chronic, and latent forms. With the intensification of dairy farming and improvements in management practices, the incidence of acute endometritis has decreased, with most cases now presenting as chronic. The main symptoms of chronic endometritis include the discharge of gray-white or yellow-brown purulent secretion from the vulva when the cow lies down. Vaginal examination reveals hyperemia and congestion of the vaginal mucosa and uterine cavity, with mucus observed on the cervix. Rectal examination reveals uterine swelling, abnormal enlargement of the uterine horns, poor uterine contractions, and uneven thickness of the uterine walls. If pyometra occurs, fluctuation of pus between the uterine horns is evident (Zhou et al.), which is consistent with the clinical presentation of cows diagnosed with endometritis in this study. In this study, the cows diagnosed with endometritis did not show obvious systemic symptoms but exhibited the discharge of white, viscous pus after lying down for a period, hanging at the vulva. When the cows stood up, large amounts of pus were discharged, attaching to the tail and drying to form dark brown, uneven lumps or sheets. Some cows did not expel uterine secretions, and discharge was only observed during rectal examination, typically as a red, viscous liquid with a foul odor. Upon rectal examination, significant fluctuation in the uterine cavity and horns was noted, with enlargement and hardening of the uterine horns, and uneven sides. The presence of polymorphonuclear leukocytes (PMNs) in uterine secretions is an indication of inflammation, which is a central pathological process in bovine endometritis. PMNs are commonly used in cytological diagnoses of uterine inflammation, with a PMN ratio ≥5% often being the diagnostic threshold (LeBlanc, 2023). In this study, after treatment, the proportion of PMNs in uterine secretions was significantly higher in the Endometritis group, reaching up to 80%, compared to the Control group. The proportion of PMNs in the uterine secretions of cows treated with the TCMF and CEF was significantly lower than that in the Endometritis group, with no significant difference between the two treatments, indicating that both treatments were effective in alleviating bovine endometritis.

In this study, uterine secretions were collected and inoculated onto blood agar plates to observe the effect of the TCMF on the uterine microbiota of dairy cows. The results showed that the Control group had fewer bacteria in the uterine secretions, with colonies appearing large and dispersed. In the Endometritis group, bacterial growth in the uterine secretions was significantly higher, but no large colonies were observed. In contrast, the bacterial count in the uterine secretions of the TCMF and CEF groups was significantly lower compared to the Endometritis group. Additionally, the TCMF group displayed colonies with hemolytic rings, similar to the effects of Timosaponin, which can treat endometritis by modulating the structure and composition of the uterine microbiota (Jiang and Xu, 2024). Based on these results, the TCMF cured 8 cows, achieving a cure rate of 80%, while CEF cured 6 cows, with a cure rate of 75%. This outcome is similar to that of Ba Qing extract, composed of Huangbai, Huangqin, Honghua, and Dangshen, which has shown comparable effects to OTC in treating bovine endometritis (Qian et al., 2020).

Metabolomics results suggest that the TCMF may alleviate the symptoms of endometritis by upregulating the synthesis and secretion pathways of cortisol. Cortisol, a glucocorticoid, has long been used as an effective drug for controlling inflammation and autoimmune diseases. Both natural and synthetic glucocorticoids are highly effective in suppressing inflammation caused by various pathogens and in preventing excessive inflammation in the body (Rao et al., 2023). Studies have shown that cortisol can inhibit the NF-κB and MAPK signaling pathways activated by LPS in RAW264.7 cells, thereby suppressing the expression of inflammatory mediators and cytokines, and reducing the cellular damage caused by inflammatory responses (Dong et al., 2018a). Additionally, cortisol inhibits the Wnt/β-catenin and PI3K/AKT signaling pathways in LPS-activated BEECs, downregulating the expression of related growth factors, which helps alleviate collagen deposition and inflammatory hyperplasia caused by the inflammatory response (Dong et al., 2018b). In this experiment, the cortisol levels in the serum of the TCMF group were significantly higher than those in the Endometritis group, suggesting that the therapeutic effect of the TCMF on bovine endometritis may involve reducing body inflammation and preventing excessive inflammatory responses.

## Conclusion

There were mainly 41 active components in *Viola yedoensis* and *Leonurus japonicus*, and they reduced the inflammation in cells and rat model. In addition, *Viola yedoensis* and *Leonurus japonicus* could decrease the levels of harmful bacteria and inflammatory markers in dairy cows, while improve the level of cortisol in the serum, the cure rate was 80%. Moreover, *Viola yedoensis* and *Leonurus japonicus* exerts therapeutic effects through TNF signaling pathway. These findings provided a theoretical basis for the development of traditional Chinese medicine in the treatment of bovine endometritis.

## Abbreviations:

ALB: albumin
BEND: bovine endometrial cells
CE: collision energies
CEF: ceftiofur hydrochloride sodium
DP: declustering potentials
HE: hematoxylin-eosin
OTC: oxytetracycline
PCA: principal component analysis
PFA: paraformaldehyde
PMNs: polymorphonuclear leukocytes
PPI: protein–protein interaction
RT-qPCR: reverse transcription quantitative polymerase chain reaction
SOD: superoxide dismutase
TCMF: *Viola yedoensis* and *Leonurus japonicus*
YMSHS: Yi mu sheng hua san
